# Supplementary material for: Does a Similar 3D Structure Mean a Similar Folding Pathway? The Presence of a C-Terminal α-Helical Extension in the 3D Structure of MAX60 Drastically Changes the Folding Pathway Described for Other MAX-Effectors from Magnaporthe oryzae
Source: Molecules. 2023 Aug 15;28(16):6068. doi: 10.3390/molecules28166068 (PMC10460046; doi:10.3390/molecules28166068)
Supplement: Supplementary file 1 [file molecules-28-06068-s001.zip › molecules-2527385-supplementary.pdf]

SUPPLEMENTARY MATERIALS

Figure S1

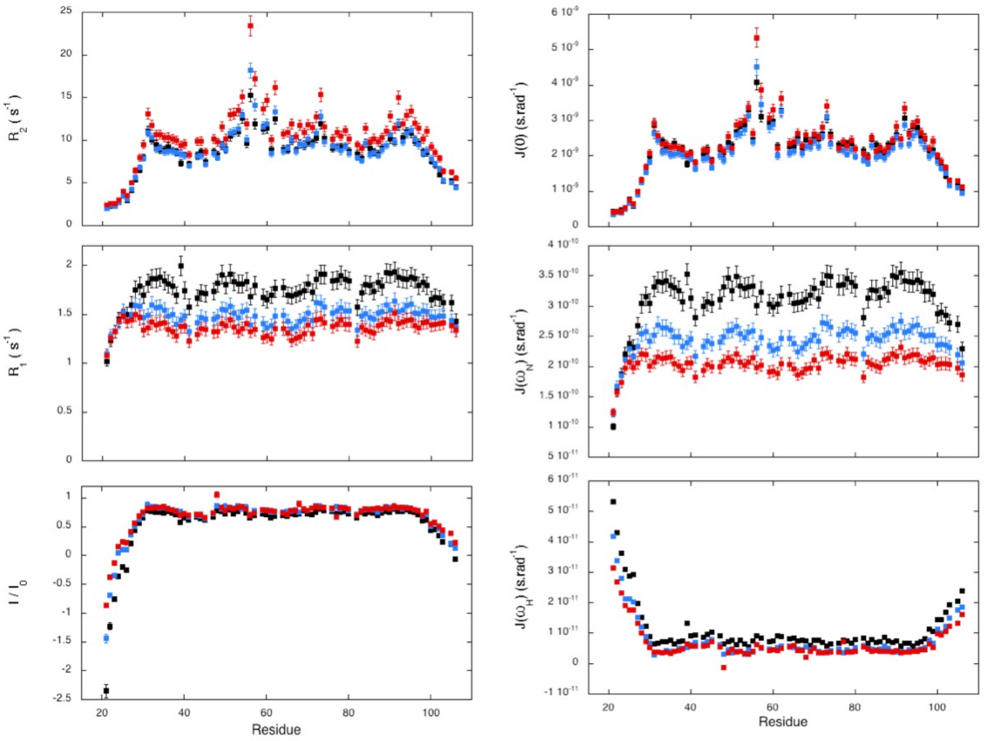

Figure S1: Multi-Field Relaxation Analysis of MAX60. Left, from top to bottom: ^{15}N heteronuclear relaxation parameters R_2, R_1 and NOE (I/I_0) measured at 14.1 T (black squares), 16.45 T (blue squares) and 18.8 T (red squares). Right, from top to bottom: spectral densities J(0), $J(\omega_N)$ and $J(\omega_H)$ obtained from the relaxation parameters measured at the three magnetic fields (same color code) through Equation [1] (Materials and Methods).

Figure S2.

A

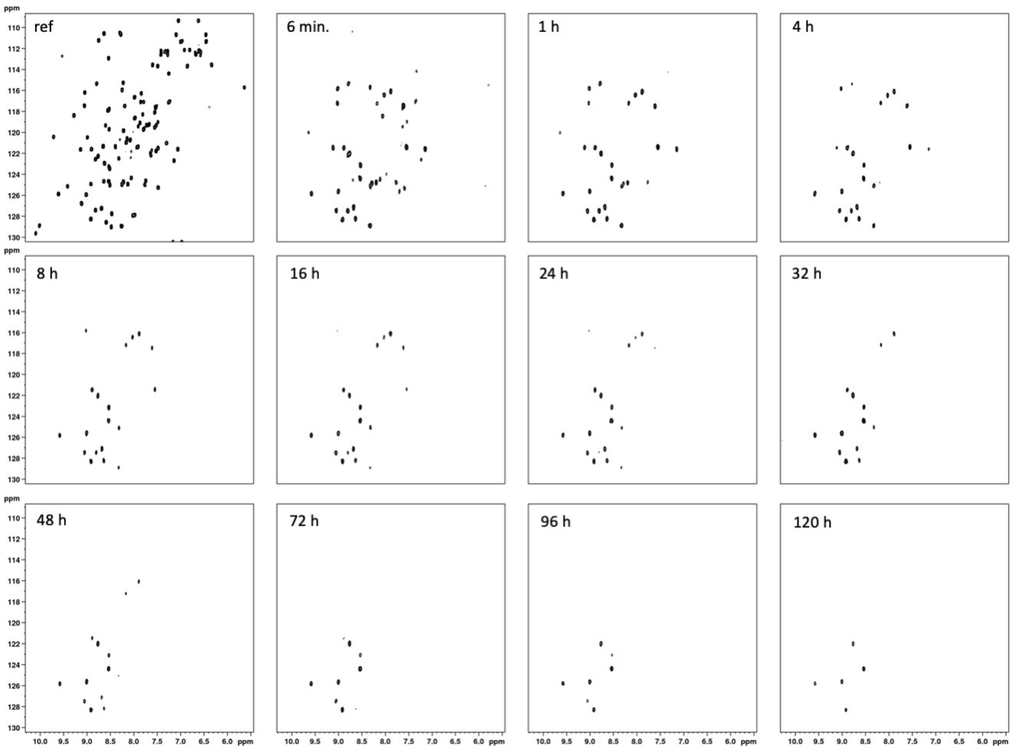

B

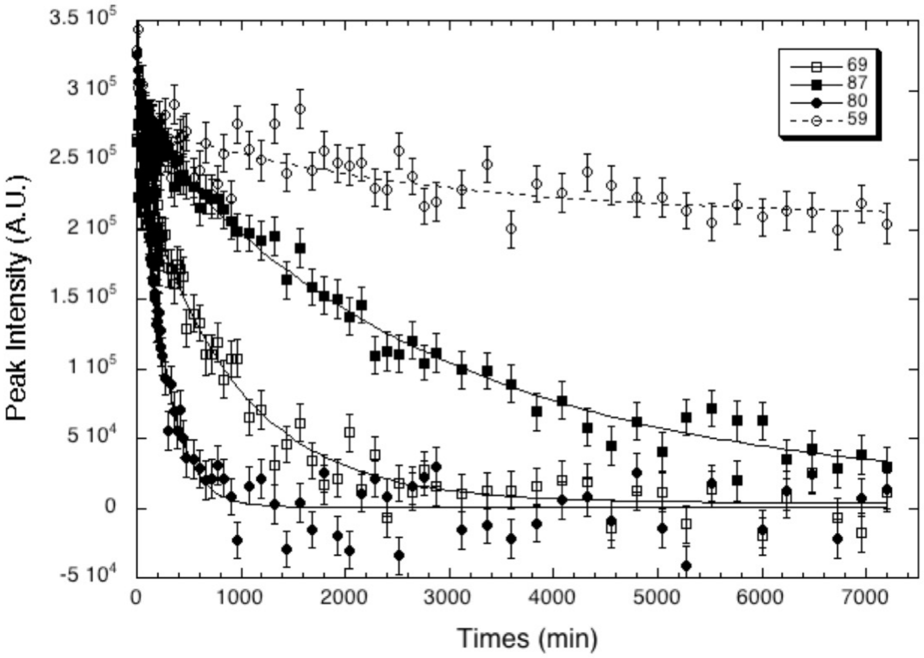

Figure S2: H/D Exchange Spectroscopy. A) Amide cross-peak intensity decays with times (insert) in a series of [^{1}H,^{15}N] HSQC 2D experiments measured on a sample of MAX60 freshly dissolved in D_2O. The reference spectrum was previously recorded in H_2O. B) Examples of fits to an exponential decay with time of the intensity of amide cross-peaks for selected residues (I59, Q69, I80 and E87).

Figure S3.

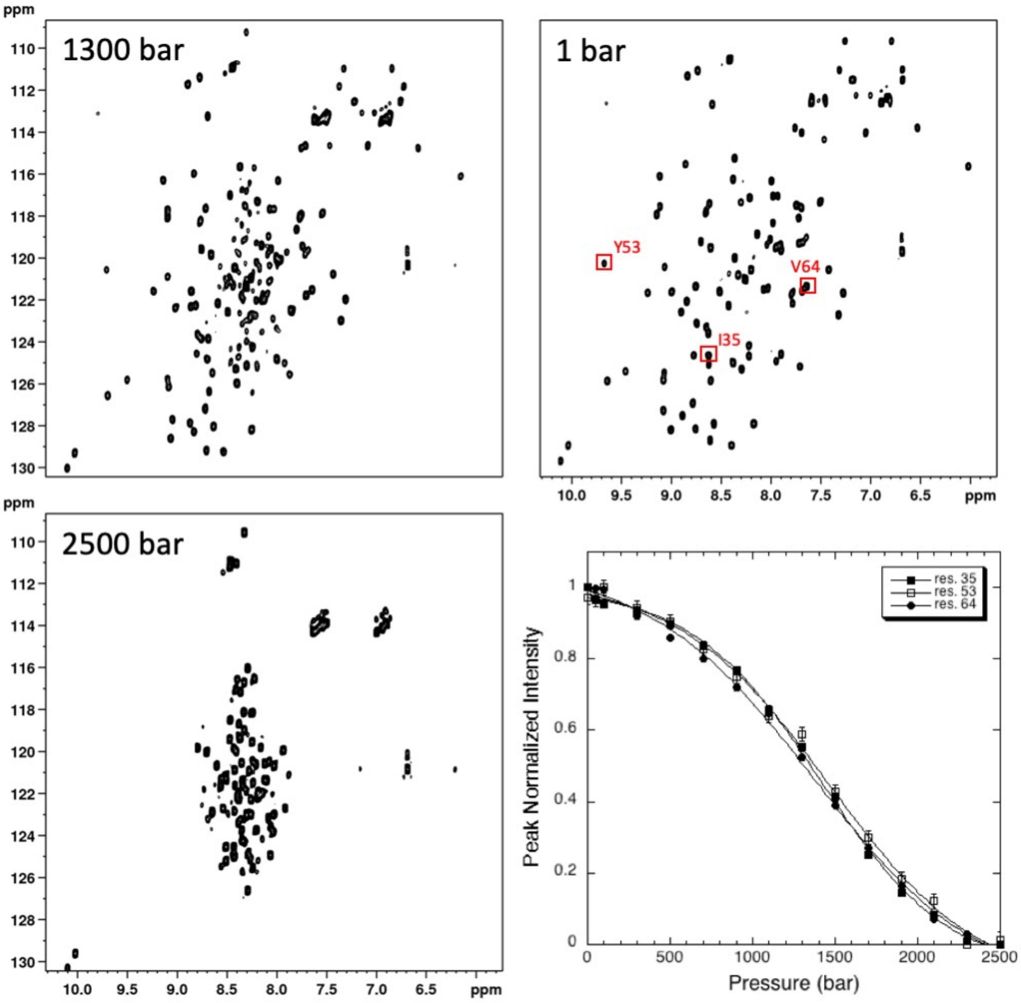

Figure S3: NMR detected pressure unfolding of MAX60 at 32°C. Examples of [^{1}H,^{15}N] HSQC recorded at 1, 1300 and 2500 bar are displayed. The last pannel shows an overlay of three (residues I35, Y53 and V64, labeled in red in the 2D spectrum recorded at 1 bar) residue-specific denaturation curves obtained from the fits of the pressure dependent sigmoidal decrease of the corresponding residue cross-peak intensities in the HSQC spectra with Eq. [5] (Materials and Methods).

Figure S4.

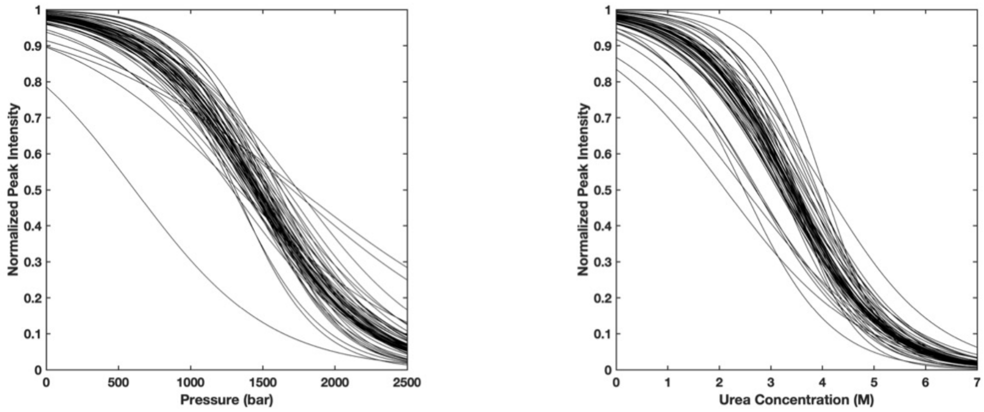

Figure S4: Overlay of the normalized residue-specific denaturation curves as obtained from the fit of the pressure-dependent (Left pannel) or the urea concentration-dependent (Right pannel) sigmoidal decrease of the residue cross-peak intensities in the HSQC spectra with Eq. [5] and Eq. [6], respectively (Materials and Methods).

Figure S5.

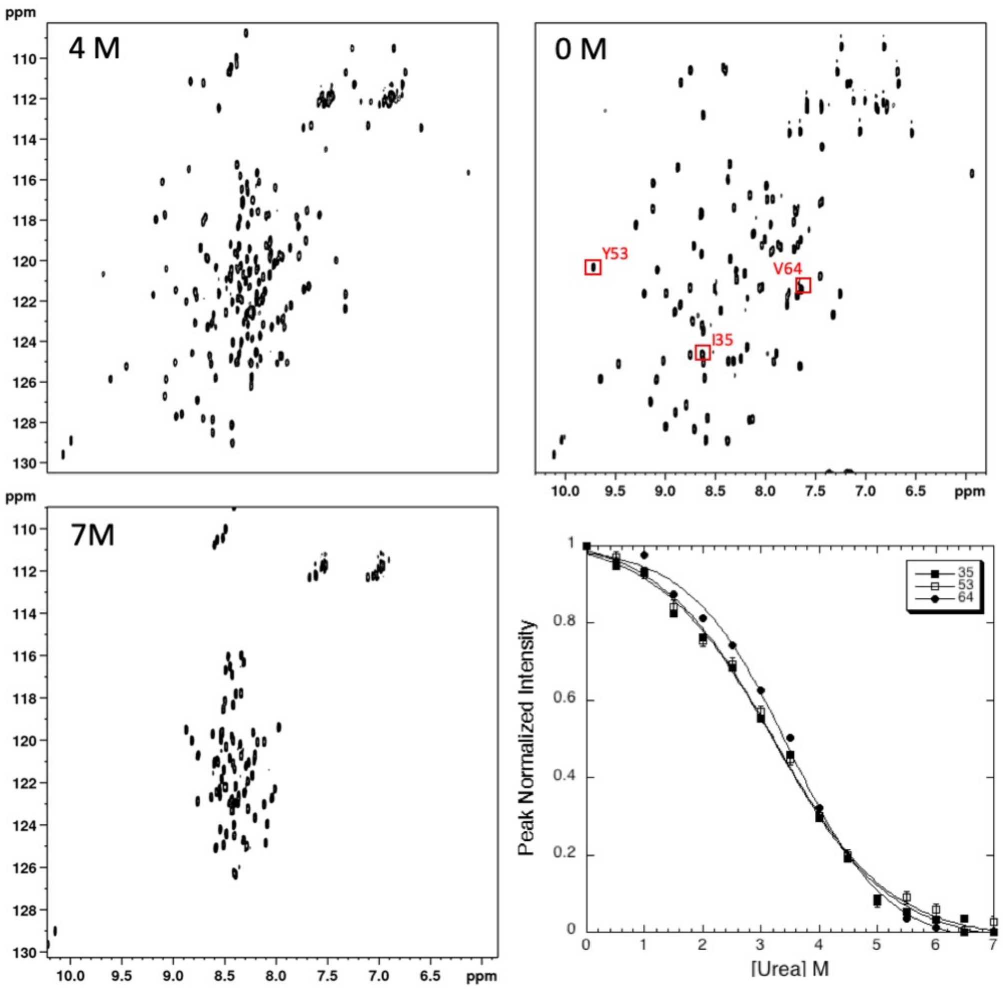

Figure S5: NMR detected chemical unfolding of MAX60 at 32°C. Examples of [^{1}H,^{15}N] HSQC at 0, 4 and 7 M urea are displayed. The last pannel shows an overlay of three (residues I35, Y53 and V64, labeled in red in the 2D spectrum recorded at 1 bar) residue-specific denaturation curves obtained from the fits of the urea concentration dependent sigmoidal decrease of the corresponding residue cross-peak intensities in the HSQC spectra with Eq. [6]] (Materials and Methods).

Figure S6.

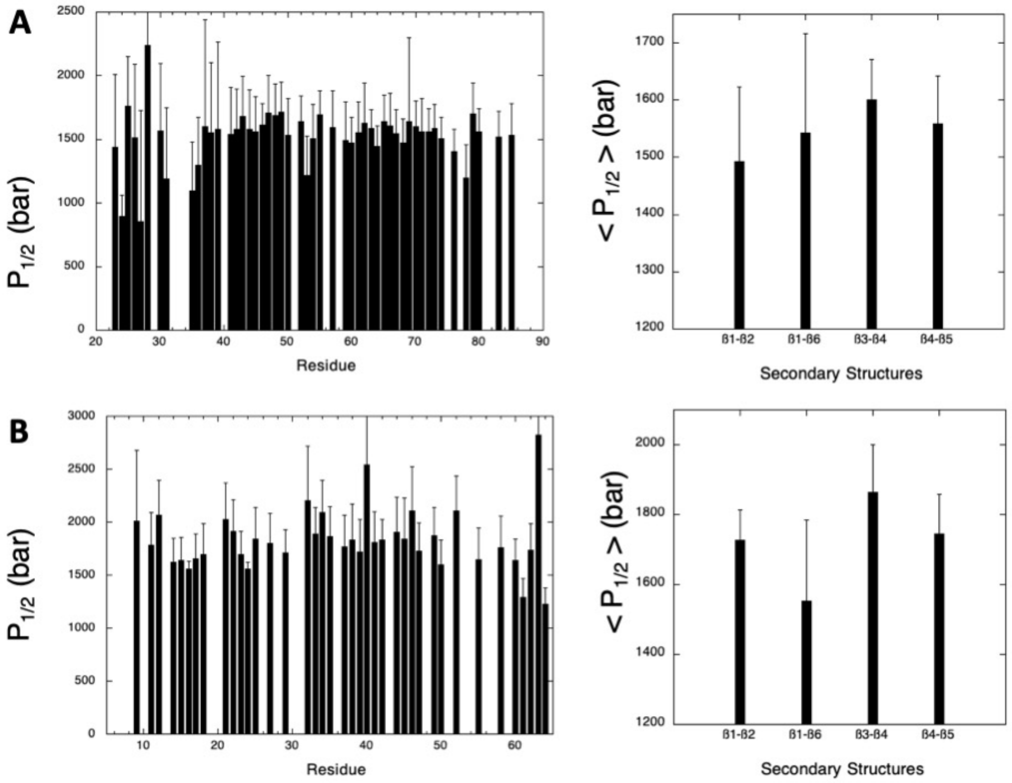

Figure S6: Half-denaturation pressure ($P_{1/2}$) values measured for AVR-Pia (A) and AVR-Pib (B). Left: half-denaturation pressure ($P_{1/2}$), obtained from the ratio $\Delta G_u^0/\Delta V_f^0$ versus the protein sequence. The thermodynamic parameters ΔG_u^0 and ΔV_u^0 have been reported previously [16]. Right: average values of the half denaturation pressure ($<P_{1/2}>$) calculated over the amide groups involved in each secondary structure element in AVR-Pia and AVR-Pib.

Figure S7.

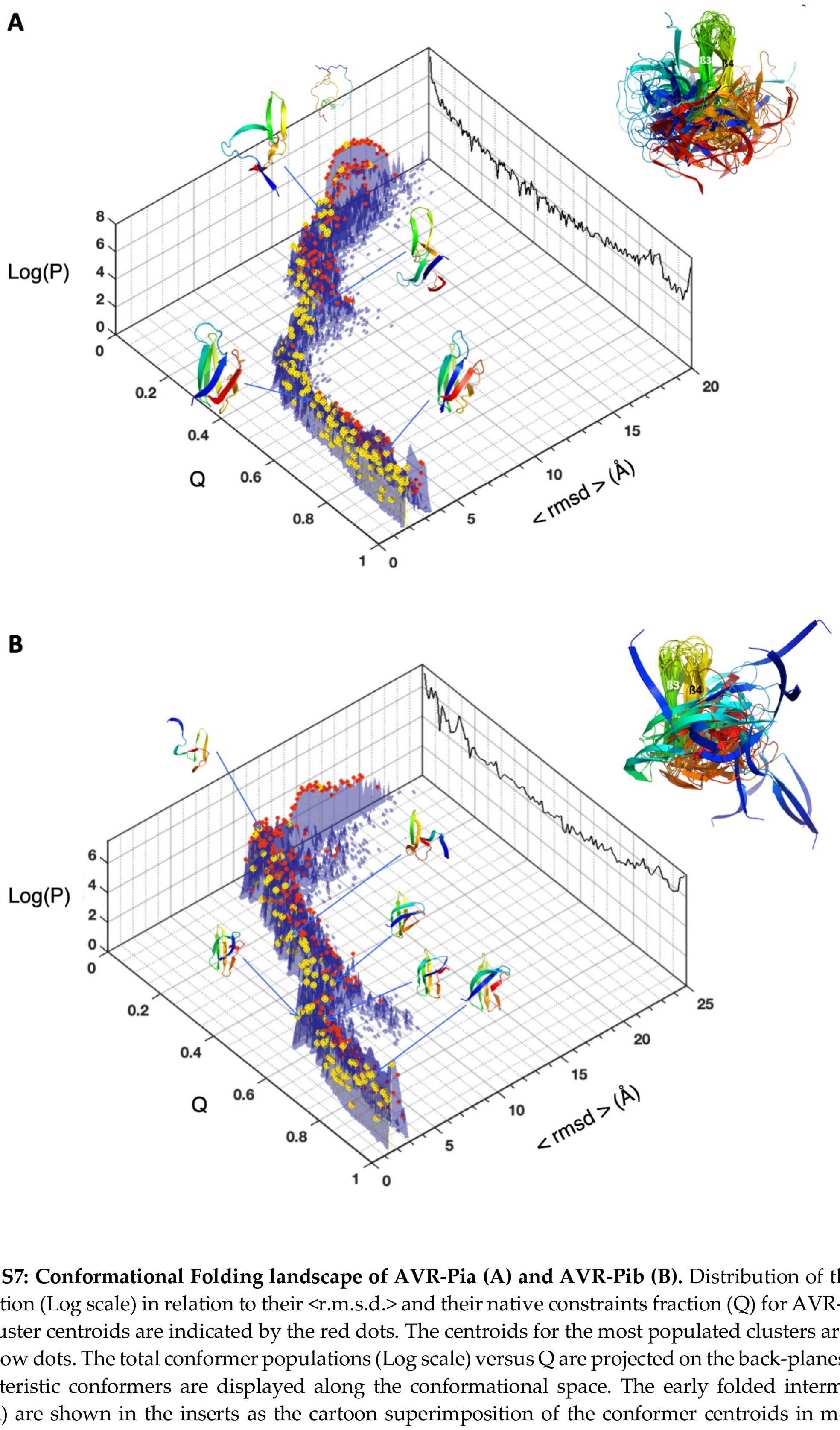

Figure S7: Conformational Folding landscape of AVR-Pia (A) and AVR-Pib (B). Distribution of the conformers population (Log scale) in relation to their <r.m.s.d.> and their native constraints fraction (Q) for AVR-Pia and AVR-Pib. Cluster centroids are indicated by the red dots. The centroids for the most populated clusters are indicated by the yellow dots. The total conformer populations (Log scale) versus Q are projected on the back-planes (black color). Characteristic conformers are displayed along the conformational space. The early folded intermediates (ß3ß4 hairpin) are shown in the inserts as the cartoon superimposition of the conformer centroids in most populated clusters within the range 0.14 < Q < 0.26. Conformers centroids are in rainbow colors from N-ter (blue) to C-ter (red) and the strands ß3 (green) and ß4 (yellow) are labelled. (Adapted from [16])

Figure S8.

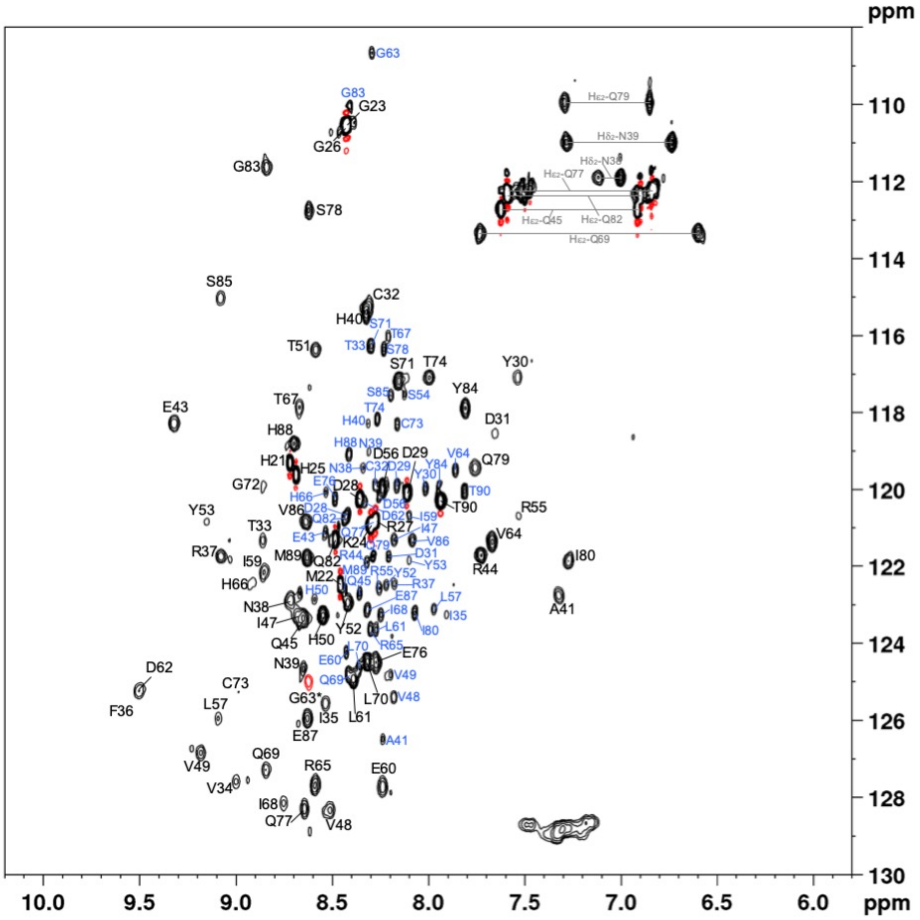

Figure S8: 2D [¹H-¹⁵N] HSQC spectra of ΔCt-MAX60 recorded at 800 MHz on a ¹⁵N-uniformly labeled protein sample dissolved in 25 mM Sodium Acetate buffer pH 4.6 at 32°C and 1 bar. Cross peak assignments are indicated using the one-letter amino acid code and number. The black and blue labels correspond to the folded and unfolded species of the protein, respectively.
